# The impact of a low-carbohydrate nutrition education program on food preferences: The correspondence between self-report consumption and supermarket purchases

**DOI:** 10.1371/journal.pone.0319503

**Published:** 2025-04-08

**Authors:** Sofia Monteiro, Georgina Pujol-Busquets, James Smith, Kate Larmuth

**Affiliations:** 1 Global Health Research Group, Kiel Institute for the World Economy, Kiel, Schleswig-Holstein, Germany; 2 Faculty of Health Sciences, Universitat Oberta de Catalunya (Open University of Catalonia, UOC), Barcelona, Catalonia, Spain; 3 MRC/Wits Rural Public Health and Health Transitions Research Unit (Agincourt), School of Public Health, Faculty of Health Sciences, University of the Witwatersrand, Johannesburg, Gauteng, South Africa; 4 Health through Physical Activity Lifestyle and Sport Research Centre, Sports Science Institute of South Africa, University of Cape Town, Cape Town, Western Cape, South Africa; 5 International Federation of Sports Medicine (FIMS) Collaborative Centre of Sports Medicine, HPALS, University of Cape Town, Cape Town, Western Cape, South Africa; 6 Division of Physiological Sciences, Department of Human Biology, University of Cape Town, Cape Town, Western Cape, South Africa; Zanjan University of Medical Sciences, Iran, Islamic Republic Of

## Abstract

There is reasonable concern that self-reported nutrition assessments do not reflect actual food choices. Yet, a correspondence between both is imperative to evaluate any intervention on food preferences. This paper makes such a comparison. It provides evidence from a low-carbohydrate nutrition education program, which is assessed with both surveys and an incentivized behavioral measure of food choice. The main result is that there is a large correspondence between survey and behavioral measures for our sample of 95 women from two historically underprivileged communities in the Western Cape, South Africa. Compared to the control, the treatment group reported a 35% lower intake from the high-carbohydrate/ ultra-processed food Red List and 60% higher intake from the low-carbohydrate whole foods Green List. The treatment group was also 40% less likely to buy anything from the Red List with a supermarket voucher. In terms of the Green List, the treatment group was significantly more likely to buy eggs, organ meat, traditional fats, avocado and fish but there was no difference in red meat and chicken, non-starchy vegetables and full cream dairy. Low-cost incentivized measures of revealed preferences can be designed to validate subjective habits, increasing confidence in the quality of evidence from nutrition intervention studies.

## Introduction

The burden of non-communicable diseases (NCDs) such as type 2 diabetes is driven to a great extent by what people eat and drink. Hence, influencing dietary behavior for disease prevention and management has become a key challenge for policymakers [1]. In response, behavioral economists have explored “How do we persuade people to eat better and lose weight?” [2] and “How do we make the healthier choice the easier choice?” [3]. There is arguably a need for evidence-based, sustainable dietary interventions that consider how people allocate their scarce resources (money, time and attention) to improve health [4]. Yet, there is currently still no consensus on how to measure dietary change accurately (in a real-world setting) adding to the challenge of improving people’s dietary preferences. While this is typically performed with self-reported surveys, there is reason to believe that what people say is not always what they do – as is, well known regarding physical activity [5], but debated with respect to dietary intake [6,7]. Our study contributes to an emerging literature on the behavioral economics of food choice [8–14].

Epidemiological observational studies, such as the much-cited Nurses’ Health Study, have significantly influenced public health policy and practice globally [15–18]. Such studies of dietary risk factors use assessments that require subjective responses (namely the Food Frequency Questionnaire (FFQ), 24-hour food recalls and food diaries) to make conclusions about the optimal diet for populations. It is understood that subjective measures of food preferences may suffer from hypothetical bias and a lack of incentive for accuracy [13,19], and in FFQs, there may be noise due to people’s inattention to what they typically eat, inability to recall fully, and social desirability bias [6,7].

We collaborated with the Eat Better South Africa (EBSA) program, a community-based non-profit organization which runs nutrition education programs in Ocean View and Atlantis; two under-resourced communities in the Western Cape. Past programs were six weeks long and involved weekly two-hour educational sessions at a central community hall with a group limited to about 30 women. These sessions aim to teach participants about nutrition, NCDs, shopping on a budget, cooking and how to access healthier foods. The program uses the Noakes Foundation’s traffic lights lists of foods which are available to the public and free to download (alternatively, see S1 Fig). Peer support via the instant messaging group is central to the program, and engagement in the group chat continues after the six-week course ends. In our previous qualitative study, the nutrition education program was evaluated using a different methodology, namely focus group discussions on women’s perceptions of the program, lifestyle choices and shopping habits [20].

Various diets are touted both in the nutrition literature and in public opinion, but the quality of evidence for them varies [21]. We posit that the behavioral measurement of food choice is a complementary tool that could increase methodological credibility of nutrition intervention studies. There is, however, no gold standard for directly assessing the validity of the FFQ [22]. Moreover, an evidence base founded on self-report survey instruments is arguably problematic [6]. Therefore, we pose the question, do revealed food preferences corroborate self-report responses in the FFQ? This is an open empirical question since in another health-related behavior, physical activity, no correlation between self-report responses and behavior was found [5]. The present cross-sectional study compares the food choices of 44 women who had taken part in a low-carbohydrate high-fat nutrition education program to 51 similar women who had not yet taken part but qualified for the program, using two complimentary diet assessment tools: purchases made with a supermarket voucher and an FFQ.

## Materials and methods

### Study design

**Fig 1:**
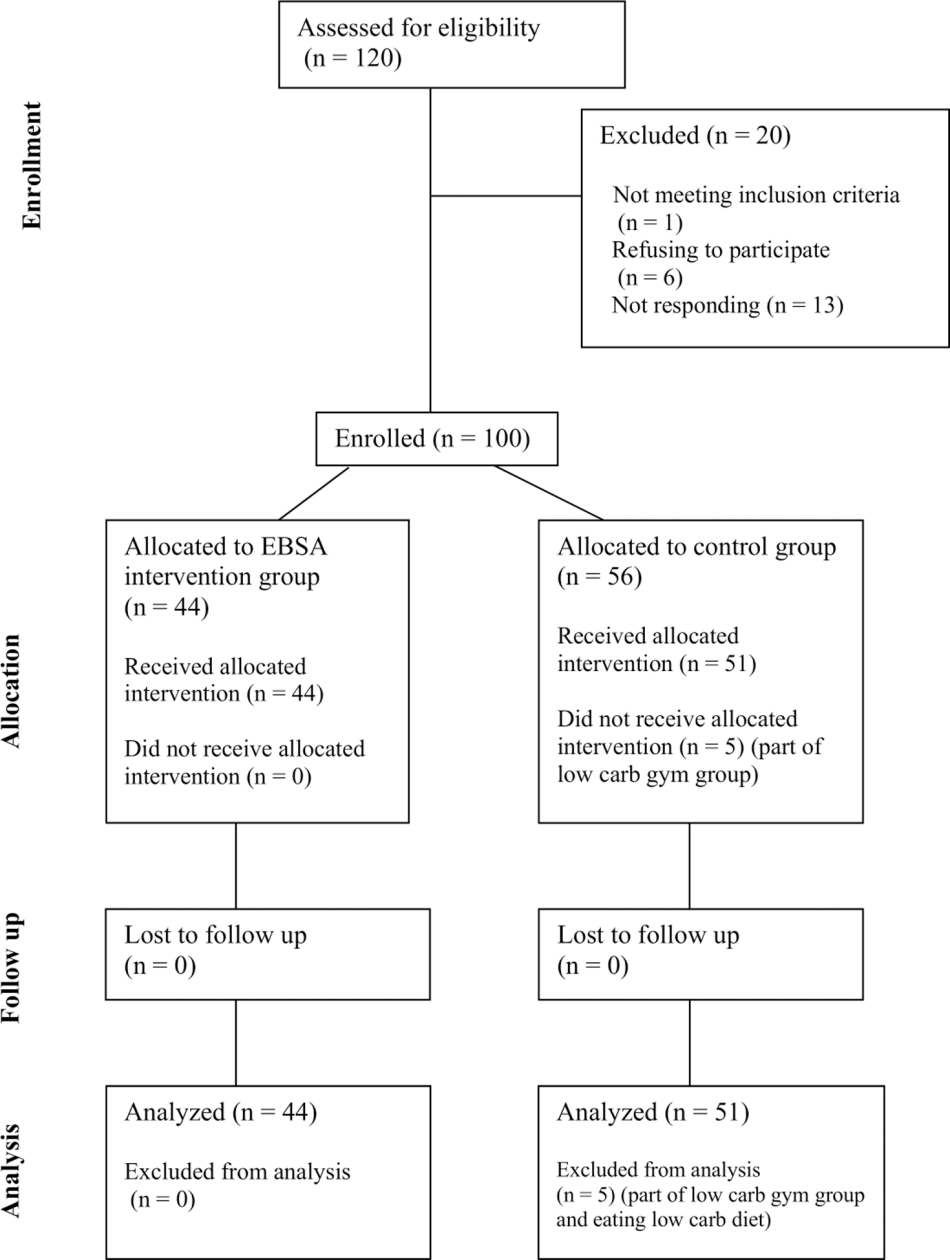
Flow diagram.

We conducted a non-randomized cross-sectional quantitative study with two groups: a treatment group of women who had already completed the EBSA program and a control group consisting of women who are eligible for future programs in the same communities. The design allowed us to identify the impact of participating in the program by controlling for observable individual characteristics. Our control group provides a convincing counterfactual to the treatment group, since the women share many similar observable characteristics. This means that, if found, a significant difference in revealed food preferences between the two groups can be attributed with a high degree of confidence to the program. Fig 1 provides a flow diagram of the study.

### Study procedures

Participation in the study took approximately 90 minutes. Each participant completed: (a) informed consent; (b) a questionnaire on socioeconomic challenges, medical conditions, food insecurity and shopping habits; (c) a task, in which they purchased food and (non-alcoholic) drink items in a local supermarket with a retail voucher and photographed their groceries and receipt; (d) a Food Frequency Questionnaire; and (e) a feedback questionnaire about their experience. Interview 1 comprised (a) and (b), while Interview 2 later in the week comprised (d) and (e).

### Ethical compliance

The study was conducted in accordance with the Declaration of Helsinki, and approved by the Human Research Ethics Committee at the University of Cape Town (HREC REF 295/2019) on 5 August 2019, as well as by the University of Cologne Faculty of Management Economics and Social Sciences Ethics Committee on 25 November 2019 (Reference: 19024SM) and the protocol amendment making the entire study remote to be compliant with Covid-19 protocols was approved 5 June 2020.

### Informed consent

Informed consent was obtained from all subjects involved in the study. Participants received study information depending on whether they were in the treatment or control group and infographics explaining the steps involved in the study via WhatsApp message. To get informed consent via telephone interview, the research assistant explained the study, sent the participant the Qualtrics survey via WhatsApp, ended the call for a couple of minutes while the participant completed it, then called them back straight away to continue the interview. If they were not able to submit the online consent form because of technological or literacy difficulties, the study information was read out loud to them, the research assistant filled out the form, and an audio recording of the participant’s verbal consent was recorded (22/95 participants).

### Inclusion and exclusion criteria

Eligible participants were: adult women; 18-69 years old; capable of providing informed consent; able to understand and speak English or Afrikaans, and not using private healthcare or private health insurance. To be eligible as a previous program participant, they had attended at least four out of six weekly sessions from one of the four previous Western Cape EBSA programs. Controls were drawn from similar communities through nomination to the study by a participant or program community coach/ambassador. This is typically how the program recruits future program participants who are relatively naïve to its Low-Carbohydrate High-Fat (LCHF) dietary advice.

### Recruitment

The recruitment period began 7 July 2020 and ended 28 August 2020. The educators and community coaches were asked to assist with recruiting women who were enrolled in previous programs, either using the WhatsApp group or by telephone. For the non-program participants, a person identified in the community was asked to help recruit women and participants were also invited to nominate a non-program woman to take part in the study. Those women interested in taking part were given the appropriate participant information sheet (i.e., treatment or control) electronically. Women were not necessarily expected to read it but were asked if they would like a researcher to contact them to explain the study. Women who communicated their interest in receiving more information about the study were contacted by a member of the research team. An infographic explaining the 10 steps to complete the study was sent electronically. A researcher explained the study, answered questions, screened for eligibility and invited interested women to take part.

## Data sources and processing

### Supermarket purchases

The participants each received a ZAR 250 (~13 USD) voucher via SMS. At the time of the study, this amount equated to about a week’s typical food shopping in our sample population. The shopping activity was held at a major local supermarket. Participants could choose from a selection of fresh produce and cupboard groceries (at current retail prices). A member of the research team explained the activity and conducted a demographic questionnaire. Each participant had a week to purchase her groceries at her convenience. An online shopping account with which the vouchers were purchased allowed the investigators to track which vouchers had been spent. Purchasing decisions were made privately in the store. The participant had access to her phone to keep track of the total cost of her shopping. When she had finished selecting items, she proceeded to the checkout. At home, she took a photo of both her groceries and the till slip, and sent them to the researcher. The researcher ticked off on a checklist with the participant’s code the types of foods she chose to buy and noted the total spent on the receipt.

### Nutrition survey

When the shopping activity was completed, the follow-up interview was booked, usually on the same day or the following day. Participants completed an interviewer-administered FFQ on a phone call with a member of the research team and were asked about foods eaten in the past four weeks. The FFQ was developed according to guidelines [22] and the South African Medical Research Council’s FFQ was adapted to include food items frequently eaten by people following a Low-Carbohydrate High-Fat (LCHF) diet and foods reportedly eaten by previous program participants. It also included standard portion sizes and frequency options. It underwent a period of pilot testing in volunteers that habitually followed a LCHF diet and was modified accordingly [23]. The FFQ data were primarily used to assess the types of foods eaten.

### Data cleaning and variable transformation

The data cleaning and analysis was conducted using Stata 15. To measure average reported dietary intake, the FFQ asked respondents how frequently they had eaten a standard portion of a particular food in the past four weeks. For example, “In the past 4 weeks, how often did you eat a slice of white bread?” to which the participant could choose one of nine fixed responses, e.g., “None”, “1 or less per month”, “2-3 per month, “1-2 per week”, 3-4 per week”, “5-6 per week”, “1 per day”, “2-3 per day” or “3 or more per day”. For the analysis, categorical variables were converted into continuous numerical variables, specifically, an estimate of the number of standard portions eaten per week. e.g., if a participant responded “1 per day”, this was converted to “7 per week”.

The food purchases that the sample of women made with a retail voucher were coded as a list of dummy variables. If a food item (e.g., bread) was purchased, this choice was captured as “1”, and “0” otherwise. The selected list of foods corresponded to the EBSA program’s traffic lights list of foods (S1 Fig) as well as the FFQ items for ease of comparison. Presentation of the reported food intake and food choices according to the traffic lights lists makes it straightforward to infer visually whether the sample of women are eating according to the program’s recommendations.

Five of the control group’s observations were excluded from the analysis because these non-program women were part of a gym group run by an EBSA community coach (Fig 1) and already eating according to the program’s dietary guidelines (S1 Fig).

A RED index score was created that added up the binary food choice variables, with a maximum score of 8 if all Red List foods were purchased. An ORANGE index score added up the relevant binary food choice variables, with a maximum score of 3 if all three Orange List foods were purchased. A GREEN index score added up the relevant binary food choice variables, with a maximum score of 8 if all eight Green List foods were purchased.

### Tests and regression models

Individual characteristics were examined to motivate that the control group can be used as a valid counterfactual to evaluate the impact of the program. The non-parametric Kruskal-Wallis H test was conducted to test for significant differences (at the conventional significance threshold of 5%) in the distributions of individual and household characteristics. The self-report dietary intake of participants was categorized into the program’s Red, Orange and Green traffic-light lists. To evaluate the impact of the EBSA program with women presumably attempting to follow the recommended LCHF diet, the types of foods eaten by the control and treatment groups were compared. If the program influenced eating habits according to its recommendations, one would expect to see fewer Red List food items and more Green List items consumed by the treatment group compared to the control group. We controlled for observable characteristics in the Ordinary Least Squares (OLS) regression analysis with robust standard errors (which also used the conventional threshold of 5% to evaluate significance). The types of food purchases of the treatment and control groups were compared. In the Linear Probability Model (LPM) regression analysis, we controlled for observable characteristics. Finally, we considered the correspondence between surveyed responses and purchasing behavior, which allowed us to make an evaluation of the validity of the FFQ. If the FFQ responses of our sample correlated well with the incentivized behavioral measure of food preferences, this would support its use as a valid instrument to assess the impact of the nutrition education program on women’s food preferences, while if the two instruments diverged this would undermine the FFQ’s credibility.

## Results

### Sample characteristics

The treatment and control groups were balanced in terms of household characteristics: number of household members, number of employed household members, shopping frequency and food insecurity (S1 Table). About half of all participants worried they would not have enough food because of lack of money in the preceding four weeks. Table 1 shows that the two groups were well balanced and similar on a number of observable individual characteristics as well. According to the most recent census conducted in 2022 the sample is representative of the ethnicity and language of our two Western Cape communities [24]. It should be noted, however, that the treatment group was significantly older (χ2(1) = 10.760, p = 0.001). The mean age for the control group was about 44 years old, while the mean age for the program group was about 51 years old. In the regression analysis, we controlled for observable characteristics.

**Table 1:** Sample characteristics.

Treatment Group	Control (n = 51)	Treatment (n = 44)
Age (years)	43.76	50.84 ^1^
Lives with partner	75%	75%
Has children	96%	93%
Buys household groceries	94%	86%
**Work status**		
Employed	53%	32% ^1^
Employed & working age	43%	38%
**Education**		
No formal schooling	0	2%
Primary school	37%	55% ^1^
High school	37%	32%
College or University	27%	9%
Post-graduate degree	2%	2%
**Health risk factors**		
Smoker (daily)	12%	7%
Alcohol (past year)	39%	41%
Elevated blood pressure	33%	52%
Blood pressure medication	16%	32%
Diabetes	12%	7%
CVD event or chest pain	4%	14%

^1^Significant p-value at the 5% level in Kruskal-Wallis H tests, which were conducted as a balance check between control and treatment groups., i.e., groups were unbalanced on this characteristic.

Almost all the women had children (mode: 2; max: 5), and 75% of the women shared the household with a partner. The women were usually responsible for groceries (χ2(1) = 1.639, p = 0.2005). On average, households had four members, with one or two members employed. A higher proportion of the control group were employed (53% compared to 32% (χ2(1) = 4.251, p = 0.0392) but there was no significant difference between the two groups at the 5% level in the proportion of working-age individuals employed, χ2(1) = 2.833, p = 0.0924. In the pooled sample, 43% had completed primary school only, 35% had completed high school and 21% higher education. The treatment group also had more participants whose highest level of education was primary school, and this difference was significant at the 5% level, χ2(1) = 5.226, p = 0.0222. However, there was no significant difference at the 5% level between the groups in basic education attainment (where primary school and high school are pooled), χ2(1) = 3.378, p = 0.0661. In South Africa, the returns to education are convex, in that the marginal rate of return is extremely high for tertiary levels of education and approaches zero for lower levels of education [25]. Thus, completing school versus not is arguably the more relevant characteristic with which to consider differences in life chances between the two groups. There were no significant differences between the groups in non-communicable disease risk factors at the 5% level, however, the control group had a marginally lower incidence of diagnosed high blood pressure at the 10% level.

### The positive effect of the program on reported food intake in the past four weeks

**Fig 2:**
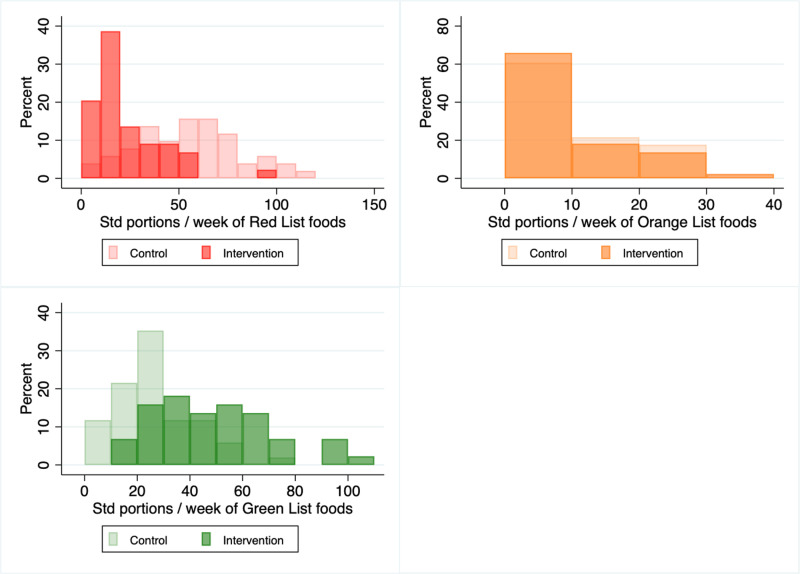
Overview of reported food intake in the past four weeks by the treatment and control group. Histograms indicate the distribution of food intake by a sample of women who have completed the EBSA program (n = 44) versus similar women who are eligible for future programs in the community (n = 51). The data are categorized by the low-carb program’s traffic lights lists of foods. See S1 Fig for detailed Green, Orange and Red Lists. Darker colored bars indicate the treatment group, while lighter bars indicate the control group. The treatment group is significantly different on Red and Green List, but not Orange List (non-parametric K-Smirnov tests for equality of distribution: **p** < 0.001).

Fig 2 shows histograms of total weekly consumption of standard portions of foods organized by the program’s traffic lights lists of foods (S1 Fig) where, according to the EBSA program, the Red List is to be avoided, the Orange List for occasional consumption, and the Green List to be eaten liberally. The Red List includes foods high in carbohydrates and seed oils such as fast food, sugar, bread, pasta, potatoes. The Orange List includes fruit, such as bananas and apples. The Green List includes eggs, unprocessed meat, unsweetened full cream dairy, green leafy vegetables. In each histogram, lighter bars indicate the distribution of the control group’s responses, darker bars indicate the distribution of the treatment group’s responses, and darkest bars show where the distributions overlap. The treatment’s total weekly consumption from the Red List is clearly left-shifted compared to the control (i.e., fewer standard portions of Red List items were consumed, K-Smirnov test: Treatment <Control, p < 0.001). In contrast, for the Green List, the distributions are reversed in favor of the program (Control <Treatment, p < 0.001). In general, for the Orange List, there is not much interesting variation between treatment and control. As we did not have particular hypotheses for the consumption from the Orange List, we focus on the Green and Red Lists in the main paper and report the Orange List results in S1 File supplementary text, for completeness.

**Fig 3:**
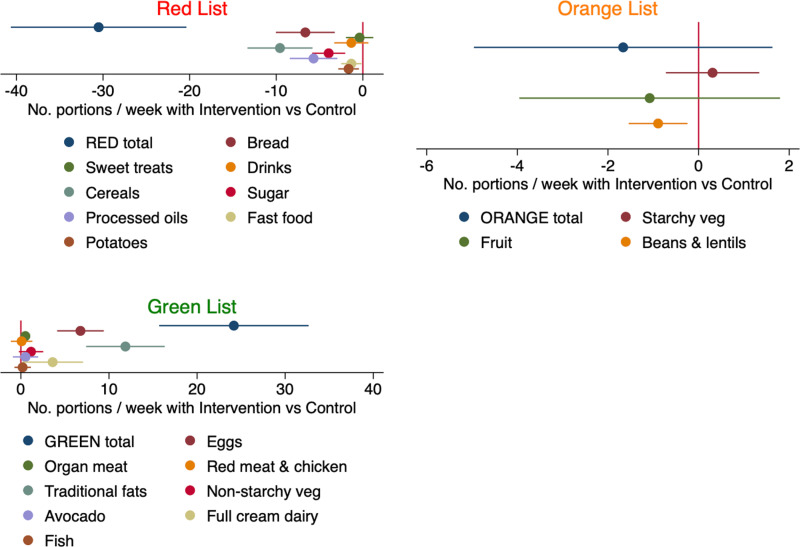
Treatment effect on the reported weekly intake of various foods in the past four weeks. Coefficient plots illustrate OLS regression models which tested if there was a significant effect of having participated in the EBSA program on reported consumption of Red, Orange and Green List foods (N = 95). Each plot represents a separate regression model. The dependent variable is number of standard portions per week reported in the FFQ. The primary explanatory variable is participation in the EBSA program vs control. Plots that cross the red vertical line where the x-axis equals zero indicate no significant treatment effect at the conventional 5% level of significance. Plots to the left (right) indicate a reduction (increase) in consumption by the treatment group relative to control group. All regressions included controls for age and highest education. Women who participated in the nutrition education program reported 35% lower Red List foods and 60% greater consumption of Green List foods than the control.

Fig 3 shows coefficient plots from OLS regressions of the number of standard portions per week on a binary variable = 1 if treatment, and 0 if control, as well as explanatory variables age and highest education. Each regression coefficient illustrated originates from a separate model. We modelled the effect of participating in the program on total weekly intake of Red, Orange and Green List foods, respectively. The effect on reported consumption of particular foods should be interpreted with caution because of multiple hypothesis testing concerns with a sample of 95 women. The OLS models in Fig 3 considered only the FFQ data. The corresponding regression results are reported in S2 Table. The treatment group reported significantly fewer total Red List portions per week, i.e., 56 in treatment versus 86 in control, a 35% reduction in unhealthy food by the treatment group, from the perspective of the program. Conversely, the treatment group reported a 60% significantly higher Green List intake, i.e., 24 weekly portions with treatment compared to 15 in control.

### The positive effect of the program on food choices with a supermarket voucher

The sustained benefit of the nutrition education program was also evident in the incentivized food choices of our sample of women. The most common response by the treatment group was choosing zero Red List foods. Only 52% of the treatment group purchased one or more Red List items with their voucher compared to 86% of the control group. On average, the control selected 2.39 (sd = 1.42) out of eight different types of Red List foods classified in the index, while the treatment selected only 1.02 (sd = 1.23). A K-Smirnov test rejected the hypothesis that the two distributions were equivalent (Treatment <Control; p < 0.001), meaning there is a statistically significant benefit of the program on the decision whether to purchase Red List food at the conventional 5% level.

In terms of the decision to choose Green List items, the treatment group purchased significantly more than the control, i.e., on average, 3.43 (sd = 1.32) versus 2.45 (sd = 1.06) out of 8, and the most common response by the treatment group was buying 4 Green List items versus 3 in the control. Almost all the women purchased at least one Green List item (Treatment: 100%, Control: 96%). A K-Smirnov test rejected the hypothesis that the two distributions were equivalent (Control <Treatment, p < 0.001), meaning there is a statistically significant benefit of the program on the decision whether to purchase Green List food, in favor of the program.

**Fig 4:**
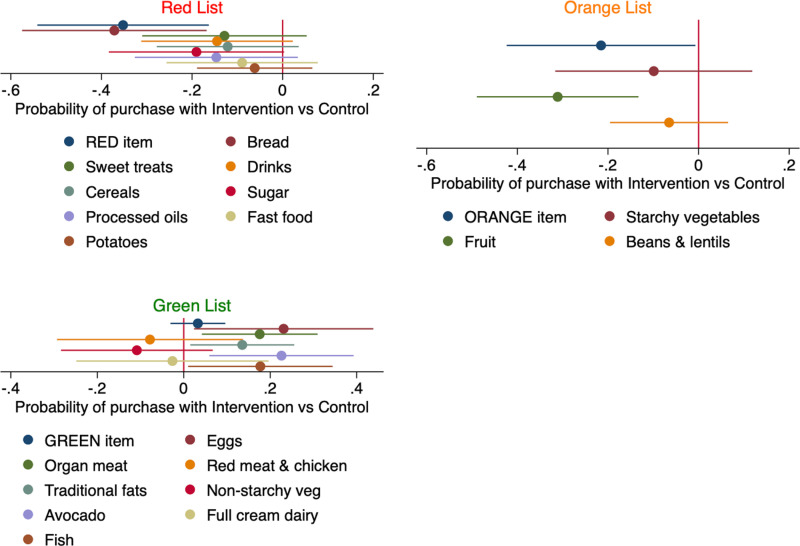
Treatment effect on the probability of purchasing Red, Orange and Green List foods. Each coefficient plot shows the treatment effect from a Linear Probability Model (LPM) regression with a dependent variable equal to 1 if a food was purchased, and 0 otherwise. The primary explanatory variable is participation in the EBSA program vs control. Plots that cross the red vertical line where the x-axis equals zero indicate no significant treatment effect at the conventional 5% level of significance. Plots to the left (right) indicate a reduction (increase) in probability of purchasing that item by the treatment group relative to control group. All regressions included controls for age and highest education. Foods were categorized by the low-carb program’s traffic lights lists (S1 Fig). Women who had participated in the program were 40% less likely to purchase anything from the Red List with a supermarket voucher and bought more of certain Green List items, including organ meat, traditional fats, avocado, fish and eggs, compared to the control group.

The coefficient plots in Fig 4 illustrate the EBSA treatment effect in regression models which used only the behavioral food choice data captured from receipts and verified by photos of groceries. Each plot is the treatment effect parameter from a Linear Probability Model (LPM) regression with a dependent variable, that was equal to 1 if a food was purchased, and 0 otherwise, and a primary explanatory variable = 1 if treatment and 0 if control. All the LPM models included control variables age and highest education. The second main result confirms the first main result: The nutrition education program influenced not only self-reported eating habits but also behavior.

The treatment group was nearly 40% less likely to purchase a Red List item with the retail voucher than the control (Fig 4). Since the treatment group was as likely as the control group to purchase at least one Green List food, we considered the separate LPM models of each of the Green List foods to learn more about the likelihood of healthy food choices from the perspective of the program. Women who had completed the program were significantly more likely to spend their voucher on five out of eight Green List items (eggs, organ meat, traditional fats (butter), avocado, fish) compared to the control group but there was no significant difference for red meat and chicken, non-starchy vegetables and full cream dairy.

### The positive relationship between incentivized choices at the supermarket and self-reported dietary intake in the past four weeks

**Fig 5:**
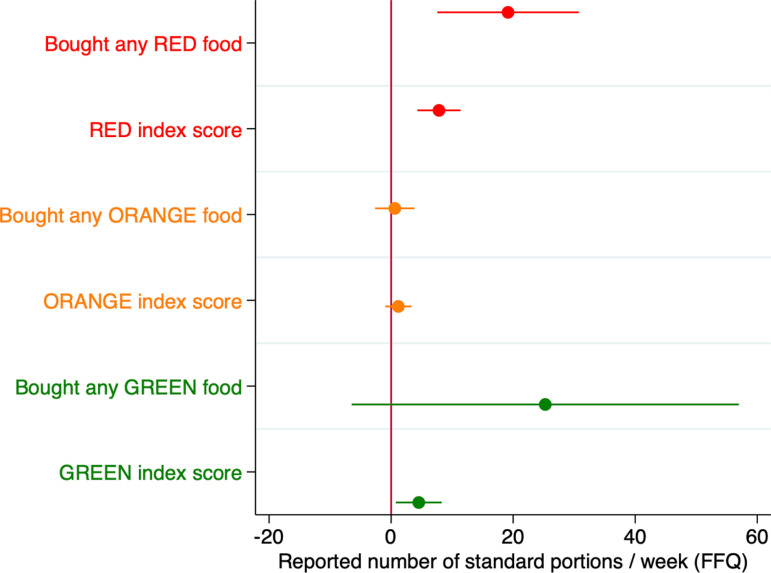
The association between purchasing behavior and self-reported dietary intake for the pooled sample. Coefficient plots are from OLS regressions that included controls for age and highest education. A significant positive association is indicated by the coefficient plot lying to the right of the vertical red line intersecting the x-axis at zero. Plots that cross the red line are not significant at the conventional 5% level. The “Bought any [RED/ORANGE/GREEN] food” variables are a binary dependent variables, while the RED/ORANGE/GREEN behavioral indices are a measure of intensity in which the dependent variable takes on discrete values (i.e., 1-8 for Green/Red List, 1-3 for Orange List). The eight coded types of Red List foods were sweet treats, cereals, processed oils, potatoes, bread drinks, sugar, and fast food. Buying each of these foods increases the RED behavior index by 1 point. The eight types of food from the Green List, which form the GREEN behavior index score are organ meat, traditional fats, avocado, fish, eggs, red meat and chicken, non-starchy vegetables, and full cream dairy. Buying each of these foods increases the GREEN behavior index by 1 point. The relationship between purchasing behavior and reported food intake tends to be positive and significant, and is particularly predictive for the RED-list.

In this section, we examine to what extent the behavioral measure validated the survey responses in the pooled sample. The coefficient plots in Fig. 5 are from six separate OLS models with robust standard errors. Starting at the top of Fig. 5, the first model “Bought any RED food” tested the hypothesis that the decision to buy a Red List food with a voucher was positively associated with reporting to have eaten more Red List food in the past four weeks, and was statistically significant. The dependent variable, total weekly intake, was regressed on an explanatory variable, RED = 1 if one or more Red List foods were purchased, and 0 otherwise. Buying any Red List food was associated with reporting 20 more Red List portions, compared to buying zero from the Red List. This is equivalent to “20 slices of white bread”, or “20 teaspoon of sugar”, for example.

Below that, the second model “RED index score”, tested the hypothesis that buying more types of Red List foods (a measure of intensity) was positively associated with higher reported Red List intake, and was also statistically significant. The eight coded types of red foods were sweet treats, cereals, processed oils, potatoes, bread drinks, sugar, and fast food. Buying each of these foods increases the RED behavior index by 1 point. A one-unit increase in the RED behavior index is significantly associated with reporting eight more standard portions of Red List foods in the survey. Overall, buying something on the Red List in the supermarket is associated with significantly greater reported Red List intake in the past four weeks.

The association between behavior and survey responses is also positive for the Green List. The eight types of food from the Green List, which form the GREEN behavior index score are organ meat, traditional fats, avocado, fish, eggs, red meat and chicken, non-starchy vegetables, and full cream dairy. Buying each of these foods increases the GREEN behavior index by 1 point. A one-unit increase in the GREEN behavior index is significantly associated with reporting five more Green List portions. The model “Bought any GREEN food” is not significant because almost everyone bought something from the Green List, but the intensity measure “GREEN index score” demonstrates the significant positive relationship between behavior and self-reported measures.

In summary, the three main results are: (1) Women who participated in the nutrition education program reported 35% lower Red List foods and 60% greater consumption of Green List foods than the control. (2) Those who had participated in the program were 40% less likely to purchase anything from the Red List with a supermarket voucher and bought more of certain Green List items, including organ meat, traditional fats, avocado, fish and eggs, compared to the control group. Finally, (3) the relationship between purchasing behavior and reported food intake tends to be positive and significant, and is particularly predictive for the Red List preferences (foods to strictly avoid, according to the program) but has little utility for the Orange List preferences (occasional consumption).

## Discussion

This paper contributes to the methodology of how to measure food preferences. Since behavior is a mechanism through which to explain observed changes in health outcomes, being able to show that a nutrition education program affects revealed food preferences, rather than hypothetical or self-reported food intake, is arguably important to demonstrate evidence of sustainable behavior change and habit formation.

We addressed three research questions: (a) Does the nutrition education program affect reported food intake, as measured by an FFQ? (b) Does the program affect food choices in the supermarket with a voucher? (c) Do incentivized food choices validate self-reported dietary intake in the past four weeks?

We found that the program not only changed reported food intake but behavior as well. The relationship between actual purchases and survey responses tends to be positive and significant, and is particularly predictive for measuring preferences for Red List food (to be avoided, according to the program). Our results on survey versus behavioral measures contrast with Prince et al. [5], which found no correlation between reported and behavioral measures in physical activity. We found that revealed preferences in the supermarket validate the survey instrument in our sample and there is utility in measuring the impact of the nutrition education program using the FFQ in this population.

This study contributes to the limited literature on behavioral economics of food choice, building on behavioral experiments such as List and Samek [9], which examined incentives for improving children’s snack choices and Charness et al. [14], which examined the impact of several non-incentivized interventions on children’s snack choices. Our study offers behavioral insights into the nutrition literature by examining the validity of a standard survey instrument for measuring food intake. Building on Cawley’s [4] economic framework for understanding eating behavior, our study empirically shows that economics offers useful insights into nutrition because it is the study of how people allocate their scarce resources of time and money. We show that the program influenced food choice behavior and how this methodology could strengthen the quality of evidence generated by impact evaluations since it is reflected in women’s survey responses about their eating habits.

With regard to limitations, the researchers’ independence from the education program and anonymity of responses was emphasized to participants to address concerns of possible experimenter demand effects. The study design put the behavioral measure first, so there was little concern about participants trying to match what they bought to their survey responses, although the reverse was possible. Participants may have desired to be consistent, but there is no reason to believe that this desire would be greater amongst our participants than in previous studies [5]. We showed how the credibility of evaluations could be strengthened by using incentivized measures of revealed food preferences when they corroborate inexpensive complementary diet assessment tools such as the FFQ.

A second limitation is that while purchased groceries are likely strongly correlated with actual food intake, it is not a measure of direct intake. Food could be wasted or given to other household members. When asked, “Are there any grocery items that you bought for someone else that you don’t plan to eat at all yourself?” less than 20% of participants answered in the affirmative. We cannot rule out that the women would not eat the treats they bought since all groceries become part of the home food environment and thus the analysis includes all purchases made with the voucher.

A third limitation of our shopping activity is the value of the supermarket voucher. What women chose to buy with ZAR 250 (13 USD) was certainly not a complete picture of their dietary intake but was nevertheless a reasonable sample of the types of foods our participants would buy given a limited budget. Allowing for unlimited expenditure would also not be a valid measure of their usual behavior. Our voucher was equivalent to about a week’s shopping value, which is less than what participants normally spend on a particular shop. Over 90% of the sample answered affirmatively to “Is what you bought similar to what you normally buy?”. The choice of ZAR 250 was taken with consideration of the research budget and achieving the sample size needed for statistical analysis. Future research could consider varying the value of the voucher as a robustness check.

A fourth limitation is the cross-sectional nature of the data. The control participants were screened to ensure they were eligible and interested in participating in future programs in their community, ameliorating concerns of sample selection bias. Future research could consider pre-post testing to examine reliability of the behavioral indices. Information spillovers to the controls cannot be ruled out and could lead to a potential underestimation of the impact of the program so the positive effect observed is a conservative estimate and the impact of the program could be even more beneficial than reported here. The main contribution is the correspondence of survey versus behavioural measures of food choice for which the non-randomized cross-sectional quantitative study design is appropriate. The nomination of control participants by a previous participant or program community coach/ambassador was pragmatic and intentional to address sample selection bias and to be consistent with how the program recruits future program participants who are not familiar with its LCHF lifestyle advice.

The data were collected in 2020 during a Covid-19 lockdown. Despite the adverse conditions, we observed a marked difference in food choice behavior by the program group compared to the control group in line with the program’s advice to choose healthy budget-friendly whole foods that are lower in carbohydrates and higher in traditional fats rather than ultra-processed seed oils. Future nutrition research could make use of complementary diet assessment tools that take into consideration revealed preferences in an ecologically valid setting, such as a local supermarket, in order to understand how people choose to allocate scarce resources of time and money when faced with lifestyle choices that impact their long-term health [4].

## Conclusion

Compared to a control group of similar women from the same two underprivileged Western Cape communities, the treatment group’s survey responses and supermarket purchases were significantly more aligned with the nutrition education program’s advice to choose budget-friendly whole foods lower in carbohydrates and ultra-processed seed oils. While only an approximation of usual eating habits, the inexpensive Food Frequency Questionnaire corresponded well with our sample’s revealed food preferences. The results suggest that when it comes to food: what she says is what she does. Future interdisciplinary research which takes a behavioral economics approach to evaluating nutrition education programs may increase confidence in the quality of evidence available for nutrition education programs to shift food preferences.

## Supporting information

S1 Fig Green List, Orange List, and Red List. Traffic lights lists of foods from the Noakes Foundation used by the Eat Better South Africa program, which detail the dietary advice. Green is to be eaten liberally, Orange for occasional consumption and Red to be avoided.
(TIFF)

S1 Table Household characteristics. (N=95).
(DOCX)

S2 Table Treatment effects on reported consumption in the past four weeks measured by the FFQ. (DOCX)

S1 File Results for Orange List reported consumption and purchases. This file provides supplementary text for the analysis.
(DOCX)
