# Supplementary figures and images for: The impact of a low-carbohydrate nutrition education program on food preferences: The correspondence between self-report consumption and supermarket purchases
Source: PLoS One. 2025 Apr 8;20(4):e0319503. doi: 10.1371/journal.pone.0319503 (PMC11978070; doi:10.1371/journal.pone.0319503)

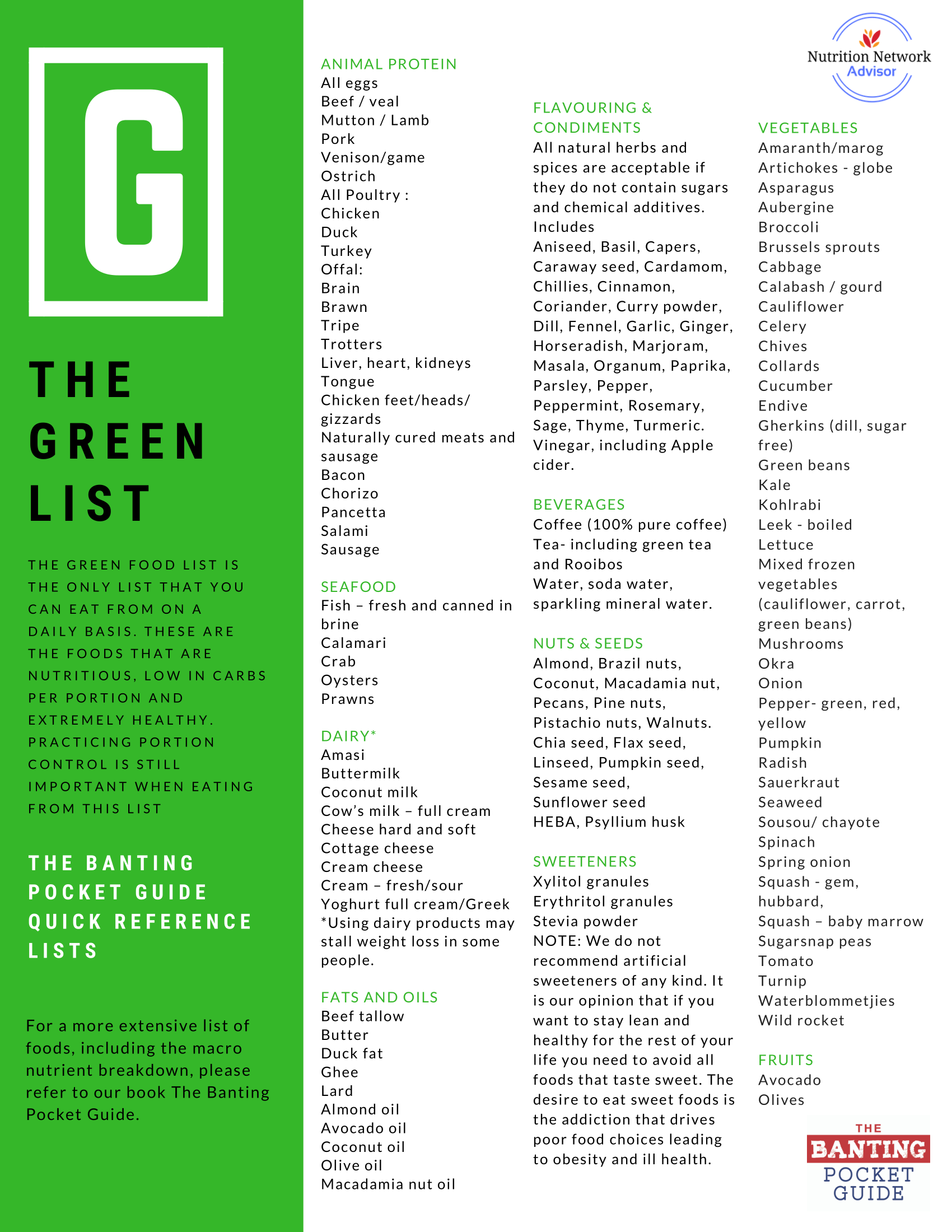

Supplement: S1 Fig — Traffic lights lists of foods from the Noakes Foundation used by the Eat Better South Africa program, which detail the dietary advice. Green is to be eaten liberally, Orange for occasional consumption and Red to be avoided. (TIFF) [file pone.0319503.s001.tiff]
