# Supplementary material for: The impact of a low-carbohydrate nutrition education program on food preferences: The correspondence between self-report consumption and supermarket purchases
Source: PLoS One. 2025 Apr 8;20(4):e0319503. doi: 10.1371/journal.pone.0319503 (PMC11978070; doi:10.1371/journal.pone.0319503)
Supplement: S1 Table — (N=95). (DOCX) [file pone.0319503.s002.docx]

**S1 Table.** Household characteristics (N = 95).

| **Treatment Group** | **Control (n = 51)** | **Treatment (n = 44)** |
| --- | --- | --- |
| Number of household members | 4.16 | 4.14 |
| Number of employed household members | 1.45 | 1.41 |
| **Grocery shopping frequency** |  |  |
| Less than once per week | 41% | 48% |
| Once per week | 41% | 48% |
| 2-3 times per week | 14% | 4% |
| Every day | 4% | 0% |
| **Food security (past 4 weeks)** |  |  |
| Worried you would not have enough food | 51% | 43% |
| Not able to eat the kinds of food you wanted | 43% | 50% |
| Not able to eat any luxury foods | 53% | 39% |
| Had to eat a limited variety of foods | 51% | 45% |
| Had to eat food you did not want to | 35% | 30% |
| Had to eat smaller meals | 45% | 43% |
| Had to eat fewer meals | 43% | 43% |
| Ever no food of any kind in your household | 12% | 7% |
| Any household member went to bed hungry | 4% | 2% |
| Any household member went 24 hrs without food | 4% | 0% |
| Affirmative answers (frequency) | 2.88 | 2.64 |

^1^ Kruskal-Wallis H tests as a balance check between Control and Treatment (EBSA Intervention) groups showed no significant differences on household characteristics at the conventional significance threshold of 5%.
