# Supplementary material for: The impact of a low-carbohydrate nutrition education program on food preferences: The correspondence between self-report consumption and supermarket purchases
Source: PLoS One. 2025 Apr 8;20(4):e0319503. doi: 10.1371/journal.pone.0319503 (PMC11978070; doi:10.1371/journal.pone.0319503)
Supplement: S2 Table — (DOCX) [file pone.0319503.s003.docx]

**S2 Table.** Treatment effects on reported consumption in the past four weeks measured by the FFQ.

|  | ***Number of portions per week*** | |
| --- | --- | --- |
| **Parameter** | **Red List^1^** | **Green List** |
| Treatment group | -30.51*** | 24.18*** |
|  | (5.11)^2^ | (4.27) |
| Age (years) | -0.49* | 0.03 |
|  | (0.24) | (0.20) |
| Highest education level | -4.93 | 5.18* |
|  | (3.08) | (2.58) |
| Constant^2^ | 86.09*** | 15.17 |
|  | (14.16) | (11.84) |
| Observations | 95 | 95 |
| R-squared | 0.04 | 0.28 |

^1^ Foods were categorized according to the Noakes Foundation’s Traffic Lights lists of foods used in the Eat Better South Africa program, which are free and publicly available online (S1 Fig).

^2^ Standard errors reported in parentheses * p-value < 0.10, ** p-value < 0.05, *** p-value < 0.01. Constant shows the intake by the control group.
