# Supplementary material for: The impact of a low-carbohydrate nutrition education program on food preferences: The correspondence between self-report consumption and supermarket purchases
Source: PLoS One. 2025 Apr 8;20(4):e0319503. doi: 10.1371/journal.pone.0319503 (PMC11978070; doi:10.1371/journal.pone.0319503)
Supplement: S1 File — This file provides supplementary text for the analysis. (DOCX) [file pone.0319503.s004.docx]

Supplementary text.

**Results: Orange List food intake and purchases**

As we did not have particular hypotheses about consumption of the Orange List foods, we provide discussion of the Orange List here for completeness. According to the Noakes Foundation’s traffic lights lists (S1 Fig), which are publicly available at thenoakesfoundation.org as well as in supplementary materials of this paper, Orange List foods are indicated only for occasional consumption once weight loss goals have been achieved. There is no significant difference in total weekly intake of Orange List foods between the two groups in the past four weeks. In the control group, 66.67% of women purchased at least one Orange List food item with her voucher, compared to 54.55% of program participants. On average, the control group selected 0.94 (sd = 0.79) out of 3 and the treatment group selected similarly 0.61 (sd = 0.62) out of 3. The modal ORANGE index score was 1 for both groups. A Kolmogorov-Smirnov test failed to reject the hypothesis that the two distributions are equivalent at the conventional level of 5% (Treatment < Control; p = 0.134). There is no significant difference between the two groups’ Orange List purchases. The LPM model “ORANGE” indicates that program participants were about 20% less likely to purchase an Orange List food. The association between behavioral and survey responses is not significant for the Orange List. Buying any Orange List food is not significantly associated with increased reported total weekly intake. There are three different types of foods in the Orange List that form the ORANGE index score we created. A one-unit increase in the ORANGE index score is not significantly associated with reporting more standard portions of Orange List foods in the survey. Overall, unlike the Red and Green Lists, buying from the Orange List is not significantly associated with reporting greater total weekly intake of Orange List foods in the FFQ.
